# Compendium of Children’s Diseases

**Published:** 1875-04

**Authors:** 


### Books Reviewed.

*Compendium of Children's Diseases: A Hand-book for Practitioners and Students.* By Dr. Johann Steiner. Translated from the Second German Edition by Lawson Fait, F. R. C. S.

The author informs us that it has been his object to make the work "a trustworthy guide to the student as well as the practitioner." The book opens with an account of the methods to be employed in examining a child. The various steps to be pursued are described briefly, but, nevertheless, in such a manner that they do not fail to make an impression.

Chapter Second is devoted to a consideration of diseases of the nervous system. both acquired and congenital, and forms a very interesting portion of the work, The following eight chapters treat of the ordinary diseases of infancy and childhood. We are forced to admire the general style of the author; he evidently knew what he had to say, and when he had said it. Nothing that is at all superfluous is found in the book, and nothing seems to be omitted which one would expect to find in a work of this character—one that is intended as a compendium simply.

As an appendix, the book contains Rules for the management of Infants, issued by the Staff of the Birmingham Hospital for Sick Children, which are of much value and are alone worth the price of the book.

